# Metal-mediated base pairs in parallel-stranded DNA

**DOI:** 10.3762/bjoc.13.265

**Published:** 2017-12-13

**Authors:** Jens Müller

**Affiliations:** 1 Westfälische Wilhelms-Universität Münster, Institut für Anorganische und Analytische Chemie, Corrensstraße 30, 48149 Münster, Germany

**Keywords:** DNA, metal-mediated base pairs, nucleic acids

## Abstract

In nucleic acid chemistry, metal-mediated base pairs represent a versatile method for the site-specific introduction of metal-based functionality. In metal-mediated base pairs, the hydrogen bonds between complementary nucleobases are replaced by coordinate bonds to one or two transition metal ions located in the helical core. In recent years, the concept of metal-mediated base pairing has found a significant extension by applying it to parallel-stranded DNA duplexes. The antiparallel-stranded orientation of the complementary strands as found in natural B-DNA double helices enforces a cisoid orientation of the glycosidic bonds. To enable the formation of metal-mediated base pairs preferring a transoid orientation of the glycosidic bonds, parallel-stranded duplexes have been investigated. In many cases, such as the well-established cytosine–Ag(I)–cytosine base pair, metal complex formation is more stabilizing in parallel-stranded DNA than in antiparallel-stranded DNA. This review presents an overview of all metal-mediated base pairs reported as yet in parallel-stranded DNA, compares them with their counterparts in regular DNA (where available), and explains the experimental conditions used to stabilize the respective parallel-stranded duplexes.

## Introduction

**Figure 1:**
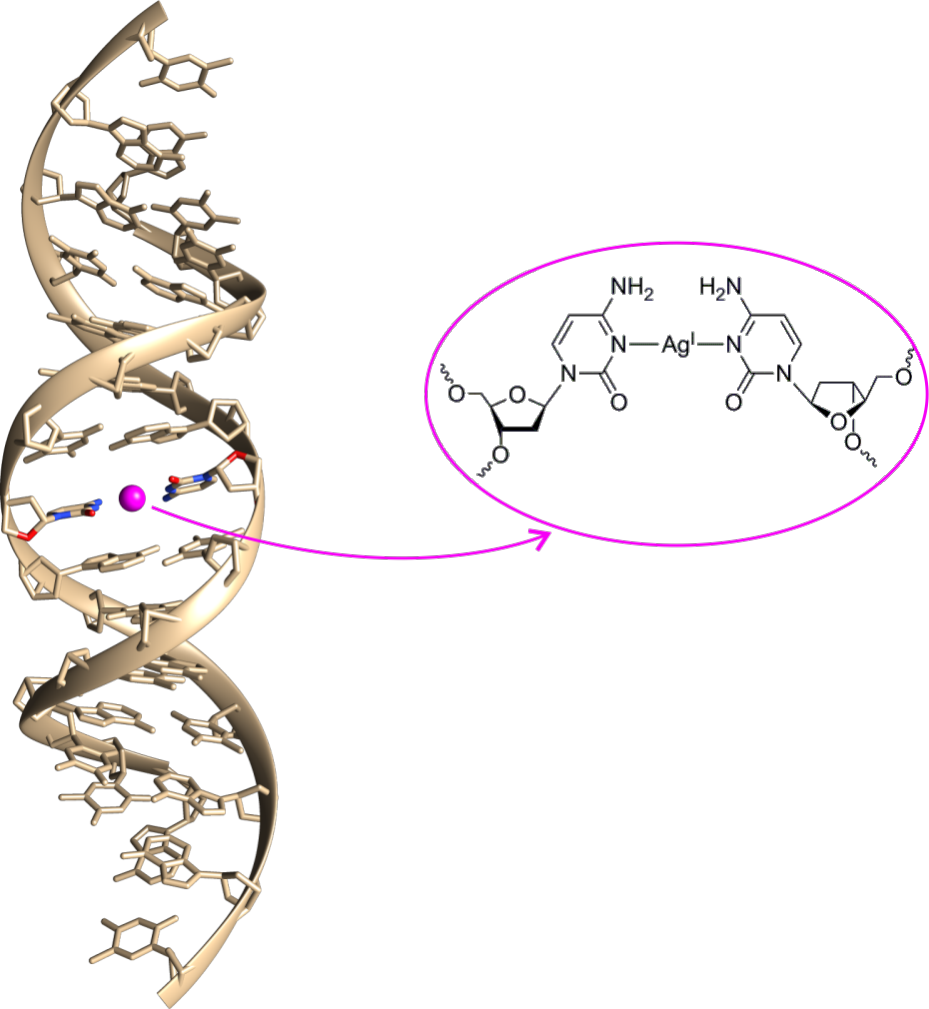
Structure of a B-DNA duplex comprising a central C–Ag(I)–C base pair. This figure was created based on the coordinates reported in PDB entry 2RVP [25].

Nucleic acids are increasingly being applied in areas beyond their original biological context, e.g., as a scaffold for the defined spatial arrangement of functional entities [1–3]. This often goes along with the formal substitution of a canonical nucleoside (or any other nucleic acid component) by an artificial one that either bears the desired functionality or contains an anchor for a postsynthetic introduction of the functional moiety [4]. The site-specific incorporation of transition metal ions is nowadays typically achieved by introducing so-called metal-mediated base pairs into the duplex. In a metal-mediated base pair, the complementary nucleobases are pairing via coordinate bonds rather than hydrogen bonds (Figure 1). Metal-mediated base pairs can be obtained from natural nucleobases such as cytosine or thymine [5]. In addition, many artificial nucleobases have been developed for an application in metal-mediated base pairing [6–7]. Structural analyses have shown that their formation is possible without major conformational changes of the nucleic acid [8], even though metal-modified nucleic acids may very well adopt non-helical topologies [9]. It is even possible to create DNA duplexes composed of metal-mediated base pairs only [10]. Possible applications of nucleic acids with metal-mediated base pairs exist in numerous fields [11]. More recently investigated areas include charge transfer in metal-modified DNA [12–14], the recognition of specific nucleic acid sequences [15–17], the creation of dynamic and switchable DNA nanostructures [18–19], and an exploitation of their processing by polymerases [20–24].

Most metal-mediated base pairs reported so far have been introduced into canonical antiparallel-stranded nucleic acid duplexes [26], even though the idea of ligand-based nucleosides has also been applied to triplexes and quadruplexes [27–30]. More recently, metal-mediated base pairs have also been investigated in the context of parallel-stranded duplexes. This review presents an overview of metal-mediated base pairs introduced into parallel-stranded duplexes so far and compares them with the corresponding base pairs in regular antiparallel-stranded DNA. In the next section, it first introduces into the concept of parallel-stranded DNA and explains different experimental approaches to enforce a parallel alignment of the complementary oligonucleotide strands.

## Review

### Parallel-stranded DNA

In canonical DNA duplexes, the complementary oligonucleotide strands are oriented in an antiparallel fashion. From a geometrical point of view, this correlates with a cisoid orientation of the glycosidic bonds in Watson–Crick base pairs. A parallel-stranded orientation of oligonucleotide strands may also occur in nature, albeit in more complex topologies such as triple helices or quadruplexes [31–32]. Nonetheless, the formation of parallel-stranded DNA duplexes can be induced in various ways, leading to a variety of non-canonical DNA duplex topologies that depend on the experimental approach taken to enforce a parallel alignment of the strands. Typically, parallel-stranded duplexes are less stable than their respective antiparallel-stranded counterparts [33–34]. This makes them of interest for the incorporation of metal-mediated base pairs, because the formation of such a base pair within an intrinsically unstable duplex is often accompanied by an exceptional thermal stabilisation, which in turn is advantageous for possible sensor applications. A feature of many base pairs in parallel-stranded duplexes is the transoid orientation of their glycosidic bonds, even though their formation is in principle also compatible with cisoid glycosidic bonds. Hence, metal-mediated base pairs that require a transoid orientation of the glycosidic bonds may be ideally generated in a parallel-stranded double helix. This section summarizes base pairing patterns established for parallel-stranded DNA duplexes in general and highlights experimental approaches feasible for the generation of such double helices.

#### Reversed Watson–Crick base pairing

**Scheme 1:**
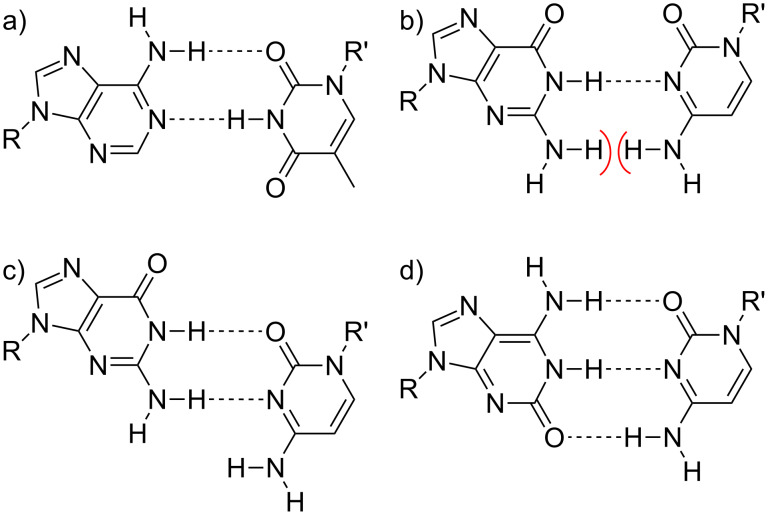
Hydrogen-bonding patterns of various reversed Watson–Crick base pairs. a) A:T; b) G:C (steric clash between opposing amino groups indicated by red hemicycles); c) G:C in an alternative geometry; d) *^i^*G:C. R, R’ = DNA backbone.

From a geometrical point of view, the simplest way to convert an antiparallel-stranded duplex with Watson–Crick base pairs into a parallel-stranded one is the formal dissociation of one of its component strands into nucleotides, the rotation of each nucleotide by 180° along the long axis of the base pair, and reconnection of the backbone of that strand. This essentially reverts the Watson–Crick base pairs to give reversed Watson–Crick base pairs [35–37]. As can be seen in Scheme 1a, the resulting A:T base pair contains two hydrogen bonds and hence can be expected to be of similar stability as is canonical counterpart. In contrast, application of the above-mentioned formalism to a Watson–Crick G:C pair leads to a base pair comprising one hydrogen bond only and in addition a destabilizing steric clash between two opposing amino groups (Scheme 1b). As a result, most reports on parallel-stranded DNA involving reversed Watson–Crick base pairs focus on A:T rich-sequences. The presence of interspersed G:C base pairs within a duplex strongly destabilizes its structure [38]. Interestingly, a slight displacement of one of the bases in a G:C pair along the short axis could enable the formation of a more stable base pair with two hydrogen bonds (Scheme 1c) [39], albeit at the cost of a backbone distortion due to the displaced positions of the glycosidic bonds. Hence, when contiguous stretches of G:C base pairs are present in a parallel-stranded duplex, thereby reducing the effect of a local backbone distortion, they are much less destabilizing. An elegant way to circumvent the low stability of a G:C base pair in parallel-stranded DNA is the use of isoguanine (*^i^*G) or 5-methylisocytosine (*^i^*C) to form *^i^*G:C or G:*^i^*C base pairs with three hydrogen bonds each (Scheme 1d) [40–41].

#### Hoogsteen and reversed Hoogsteen base pairing

**Scheme 2:**
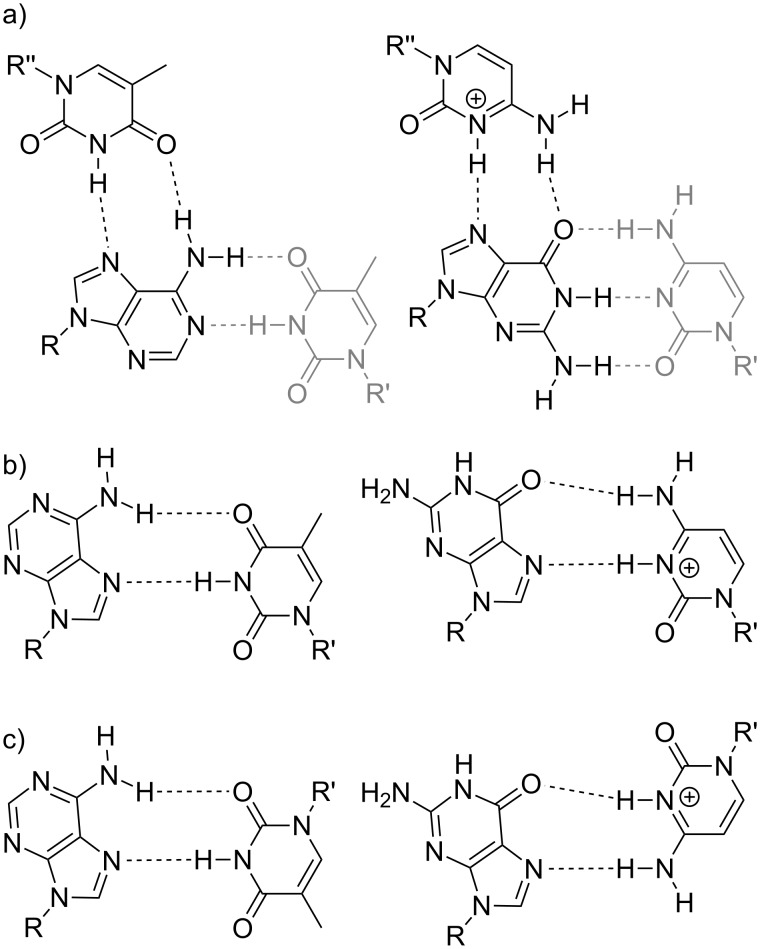
Various hydrogen-bonding patterns. a) T:A:T and CH^+^:G:C base triples indicating how T:A and CH^+^:G Hoogsteen-type base pairs are formally derived from a pyrimidine:purine:pyrimidine base triple. The pyrimidine residue involved in the Watson–Crick pairing within each triple is indicated in grey. b) Hoogsteen-type A:T and G:CH^+^ base pairs. c) Reversed Hoogsteen-type A:T and G:CH^+^ base pairs. R, R’, R’’ = DNA backbone.

Hoogsteen and reversed Hoogsteen base pairing can commonly be found in triple helices. The triplex most relevant in the context of this review is the pyrimidine:purine:pyrimidine triplex, where each base triple formally comprises a regular Watson–Crick base pair and an additional pyrimidine residue hydrogen-bonded to the central purine moiety via its Hoogsteen edge. Conceptually, this Hoogsteen-bonded part of the triplex represents a duplex of its own. Scheme 2a indicates how the A:T and G:CH^+^ Hoogsteen base pairs are formally derived from the respective base triples. As can be seen, the cytosine residue needs to be protonated to engage in this hydrogen-bonding pattern. Based on the p*K*_a_ value of a cytosine residue within an oligonucleotide single strand of about 4.3 [42], it can be anticipated that this base pair is ideally stabilized under slightly acidic conditions. However, triple helices including a protonated cytosine are stable under physiological conditions, too. In this context, the apparent p*K*_a_ value of a cytosine moiety within a CH^+^:G:C triple in a triplex was reported to amount to 6.7 [43]. Hence, while preferring slightly acidic conditions, Hoogsteen base pairs may also be stable at near-neutral pH values.

When considering a duplex comprising Hoogsteen-type base pairs, the correlation between the relative orientation of the glycosidic bonds (cisoid vs transoid) and the relative orientation of the oligonucleotide strands (parallel vs antiparallel) is rather complex. As can be derived from several calculated or experimental duplex and triplex structures, a parallel strand orientation is adopted for cisoid Hoogsteen base pairing (Scheme 2b) when both nucleotides involved in the base pair adopt an identical glycosidic bond conformation, i.e., when both are oriented *anti* or both are oriented *syn* [44–46]. If they adopt opposing glycosidic bond conformations, an antiparallel strand orientation results [46–51]. The opposite is found for the transoid reversed Hoogsteen base pairing (Scheme 2c). Here, an identical glycosidic bond formation correlates with an antiparallel strand orientation [46,50–51], whereas a parallel arrangement of the strands results from opposing glycosidic bond conformations [46]. It needs to be noted that these correlations are derived from base pairs and triples comprising canonical purine and pyrimidine nucleobases only. In particular, it is assumed that both Hoogsteen and reversed Hoogsteen pairing involve the Hoogsteen edge of one purine residue and the Watson–Crick edge of the complementary pyrimidine or purine moiety. Artificial base pairs involving two purine entities facing each other via their respective Hoogsteen edge (vide infra, Scheme 8b) need to be treated differently, as this additional structural change leads to a change from parallel-stranded to antiparallel-stranded (and vice versa) in the above-made correlations. These general considerations on how the type of hydrogen-bonding pattern, the orientation of the glycosidic bonds (*syn* vs *anti*) and their relative position (cisoid vs transoid) correlates with the relative strand direction of the oligonucleotide chains are also known as Westhof’s rule [52].

#### Chimeric base pairs of α- and β-deoxyribonucleosides

**Scheme 3:**
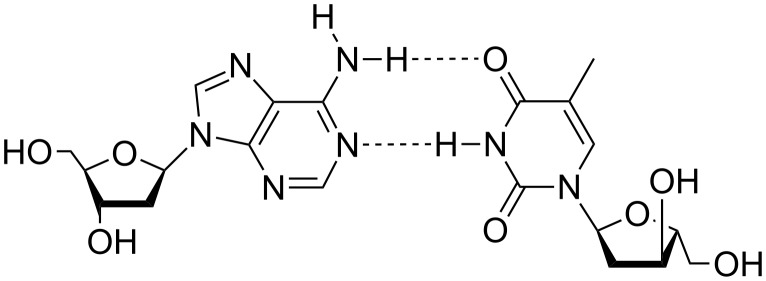
Representation of a β-dA:α-dT base pair.

Another possibility to create parallel-stranded duplexes is the use of α-anomeric nucleic acids. These oligonucleotides are formally derived via an inversion of the configuration at the C1’ position of the deoxyribonucleoside. When pairing an oligonucleotide comprising α-deoxyribonucleosides with an oligonucleotide consisting of canonical β-deoxyribonucleosides, a parallel-stranded duplex is formed [53]. Due to the reversal of the strand polarity and the concomitant inversion of the configuration, this duplex contains cisoid Watson–Crick base pairs (Scheme 3) [54–55]. Transoid reversed Watson–Crick base pairs can be obtained by introducing individual α-deoxyribonucleosides into a regular antiparallel-stranded duplex of β-deoxyribonucleosides [56].

### Mononuclear metal-mediated base pairs

**Scheme 4:**
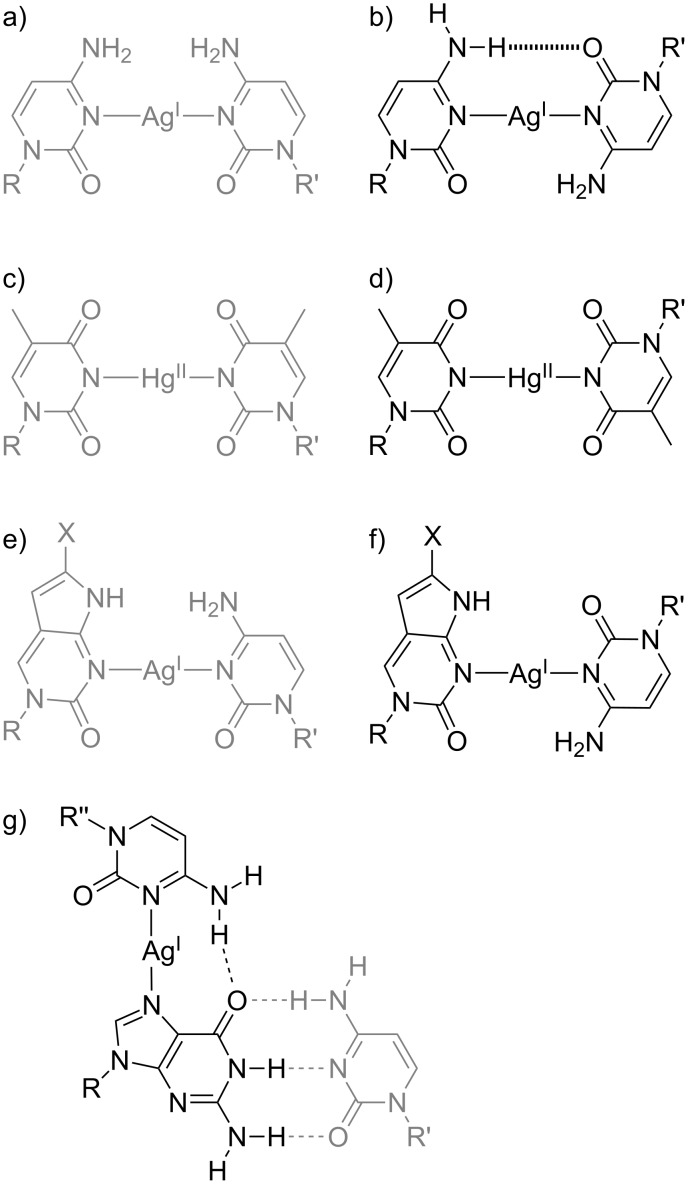
Representation of mononuclear metal-mediated base pairs involving canonical pyrimidine nucleobases. Base pairs within an antiparallel-stranded context are displayed in grey, whereas base pairs in a parallel-stranded sequence alignment are shown in black. a, b) C–Ag(I)–C [59–60]; c, d) T–Hg(II)–T [59]; e) ^X^PC–Ag(I)–C (X = CH_3_, 2-pyridyl, 3-pyridyl) [61–62]; f) ^X^PC–Ag(I)–C (X = 2-pyridyl, 3-pyridyl) [62]; g) C–Ag(I)–G:C base triple [63]. R, R’, R’’ = DNA backbone.

One of the first metal-mediated base pairs investigated both in parallel- and antiparallel-stranded DNA duplexes is the C–Ag(I)–C pair. The geometry of this base pair within regular B-DNA is depicted in Scheme 4a. It has been unambiguously proven by experimental structure determinations [10,25]. A comparison of this cisoid base pair with its transoid counterpart (Scheme 4b) suggests that the latter geometry may be additionally stabilized by a synergistic hydrogen bond. Indeed, computations indicate that the transoid base pair is favoured by 7.6 kcal mol^−1^ in the gas phase [57]. It was found to be slightly asymmetric with an N–Ag(I)–N angle of 161.7°. This asymmetry contrasts that of the symmetric hemiprotonated CH^+^:C base pair known from i-motif structures and is the result of the larger size of the Ag(I) ion compared with a proton. Hence, only one rather than two hydrogen bonds is formed. This was corroborated by a different theoretical study for a solvated (aquated) transoid C–Ag(I)–C base pair [58]. Experimentally, formation of a transoid C–Ag(I)–C base pair was achieved in two independent manners. In the first report, base pairing of the surrounding canonical base pairs in a reversed Watson–Crick pattern was achieved by covalently linking the complementary strands, fixing them in a parallel-stranded fashion [59]. The second example for a transoid C–Ag(I)–C base pair involves the use of an α-deoxycytidine residue introduced into a regular B-DNA duplex [60]. Table 1 lists the melting temperatures of the respective duplexes. Interestingly, the transoid C–Ag(I)–C base pair was found to exert a larger stabilizing effect than the corresponding cisoid C–Ag(I)–C pair in the latter example only. It is tempting to speculate that the covalent linkage used in the first study additionally influences the duplex stability, leading to a decreased stabilizing effect of the metal-mediated base pair. This hypothesis is corroborated by the fact that the transoid T–Hg(II)–T base pair (Scheme 4d) is likewise less stabilizing than the corresponding cisoid pair (Scheme 4c) when covalently linked duplexes are considered (Table 1) [59].

**Table 1:** Increase in melting temperature Δ*T*_m_ of duplexes bearing C–Ag(I)–C or T–Hg(II)–T base pairs upon formation of that metal-mediated base pair.

Base pair	Δ*T*_m_ [°C]		Nucleic acid	Type of stabilization	Ref.

	Cisoid	Transoid			

C–Ag(I)–C	+13 °C	+8 °C	DNA	covalently linked duplex	[59]
C–Ag(I)–C	+7.5 °C	+15.0 °C	DNA	α-deoxyribonucleoside	[60]
C–Ag(I)–C	+5.5 °C	+12.0 °C	DNA/RNA hybrid	α-deoxyribonucleoside	[60]
T–Hg(II)–T	+9 °C	+6 °C	DNA	covalently linked duplex	[59]

Without the constraint of being incorporated in a nucleic acid duplex comprising Watson–Crick or reversed Watson–Crick base pairs [64], additional geometries may be adopted by the C–Ag(I)–C base pair. A recent computational study on a duplex bearing Ag(I)-mediated base pairs formed from a d(CC) dinucleotide indicates significantly tilted nucleobases, leading to a conformation in-between cisoid and transoid [65]. Such an arrangement was later found in the crystal structure of an Ag(I) complex of the model nucleobase 1-hexylcytosine as well as in a non-canonical DNA structure [9,66].

Pyrrolocytosine (PC) represents a fluorescent analogue of cytosine that still retains the base pairing properties of its parent nucleobase [67]. Accordingly, its application in metal-mediated base pairing was probed, too. An initial report on the ^Me^PC–Ag(I)–C base pair (Scheme 4e) within regular B-DNA did not include any data on the stabilizing effect of the Ag(I) ion coordination but unequivocally confirmed metal-mediated base pair formation via the quenching of the intrinsic fluorescence of ^Me^PC [61]. The ^2Pyr^PC–Ag(I)–C and ^3Pyr^PC–Ag(I)–C base pairs (Scheme 4e,f) were investigated both in antiparallel-stranded and in parallel-stranded DNA. For both base pairs, the increase in melting temperature *T*_m_ upon formation of the metal-mediated base pair in parallel-stranded DNA slightly exceeded that observed for the antiparallel-stranded duplex (Δ*T*_m_ ≥ 6 °C vs Δ*T*_m_ = 5.5 °C) [62]. Even though this difference is not significant, it may be assumed that it is the result of one synergistic hydrogen bond, just like in the case of C–Ag(I)–C. In this study, the parallel-stranded alignment of the duplex was achieved by enforcing reversed Watson–Crick base pairs via the use of G:*^i^*C and *^i^*G:C base pairs.

One metal-modified nucleic acid has been reported with a C–Ag(I)–G pair in which the Ag(I) ion binds to the guanine residue via its Hoogsteen edge [63]. As mentioned above, Hoogsteen-type duplexes may be considered an excerpt from a pyrimidine:purine:pyrimidine triplex. In fact, the reported C–Ag(I)–G pair was essentially a component of a C–Ag(I)–G:C base triple within a triple helix, in which the proton of a CH^+^:G:C triple was formally replaced by an Ag(I) ion [63].

**Scheme 5:**
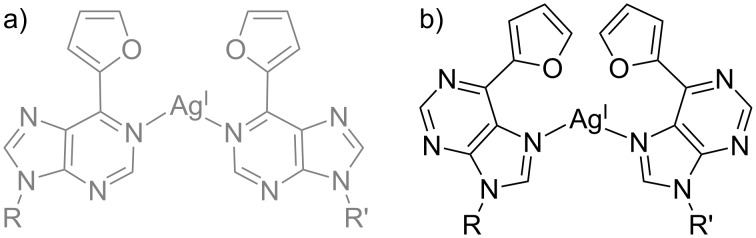
Proposed base pairing patterns of FP–Ag(I)–FP, involving a) the Watson–Crick edge (antiparallel-stranded) and b) the Hoogsteen edge (parallel-stranded) of the purine derivative [68–69]. R, R’ = DNA backbone.

In addition to the canonical pyrimidine nucleobases such as cytosine and thymine or pyrrolocytosine as a derivative thereof, 6-furylpurine (FP) was reported as an artificial purine derivative for metal-mediated base pairing. When introduced into a regular antiparallel-stranded sequence context, the thermal stabilization upon incorporation of Ag(I) was rather low (Δ*T*_m_ = 2 °C) and could not be unequivocally distinguished from unspecific binding to the canonical nucleobases [68]. However, when the FP–Ag(I)–FP base pair was incorporated into a parallel-stranded DNA duplex of the same sequence, a significant stabilization of almost 15 °C was observed [69]. In this study, a parallel-stranded orientation of the duplex was achieved by enforcing Hoogsteen base pairing via the selection of a low pH of 5.5. The strong preference for a parallel strand alignment was explained by comparing the proposed base pairing patterns for antiparallel-stranded DNA (Scheme 5a) and parallel-stranded DNA (Scheme 5b) [69]. While the relative location of the glycosidic bonds shows an enormous discrepancy between the Ag(I)-mediated Watson–Crick pair and its surrounding canonical base pairs (Δ > 2.7 Å), a perfect match was found for the Hoogsteen geometry (Δ = 0.01 Å). Hence, despite the fact that the Ag(I)-mediated Watson–Crick pair is more stable than the Ag(I)-mediated Hoogsteen pair by 15.3 kcal mol^−1^, the Ag(I)-mediated Hoogsteen base pair displays a very favourable geometry and perfectly fits the steric requirements of the parallel-stranded duplex geometry.

**Scheme 6:**
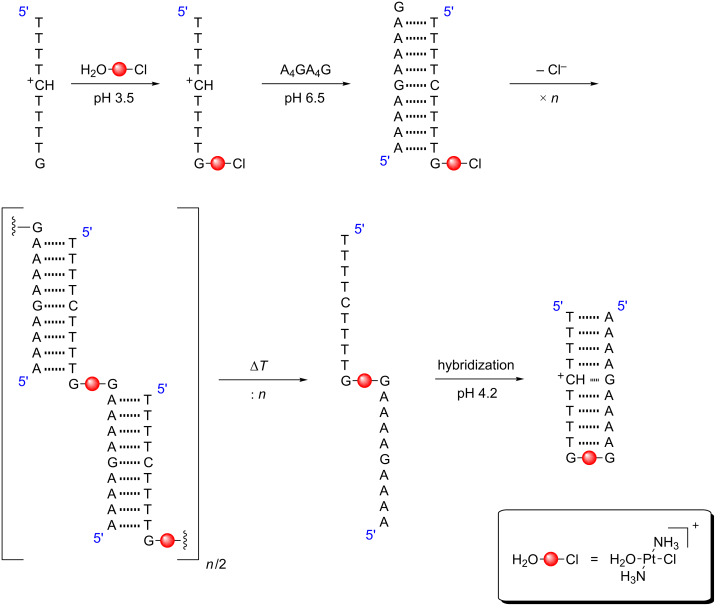
Reaction pathway towards a parallel-stranded DNA duplex bearing a G–Pt(II)–G base pair. Adapted from reference [70].

Finally, a Pt(II)-mediated base pair has been reported in which a G–Pt(II)–G crosslink enforces a parallel strand orientation [70]. The preparation of this base pair was very distinct from the procedure commonly applied for the generation of Ag(I)- or Hg(II)-mediated base pairs [11], because Pt(II) reacts under kinetic control and has a high affinity for all canonical nucleobases [71]. As shown in Scheme 6, a single-stranded pyrimidine sequence with a terminal guanine residue was initially platinated with the monoaqua species of transplatin, i.e., with *trans*-[PtCl(NH_3_)_2_(OH_2_)]^+^. As a result of the reaction conditions (pH 3.5), the platination selectively took place at the sole guanine residue. In the second step, a complementary oligonucleotide was added to form an antiparallel-stranded duplex with a dangling guanine moiety at each 3’ terminus, one of them being monofunctionally platinated. As all other nucleobases were involved in base pairing, the next platination proceeded after slow dissociation of the remaining chlorido ligand in an intermolecular fashion. Once this slow reaction was completed, brief heating to interrupt the hydrogen-bonded base pairs and subsequent slow annealing under slightly acidic conditions favoured the formation of a parallel-stranded duplex with Hoogsteen base pairing and one G–Pt(II)–G base pair.

### Dinuclear metal-mediated base pairs

**Scheme 7:**
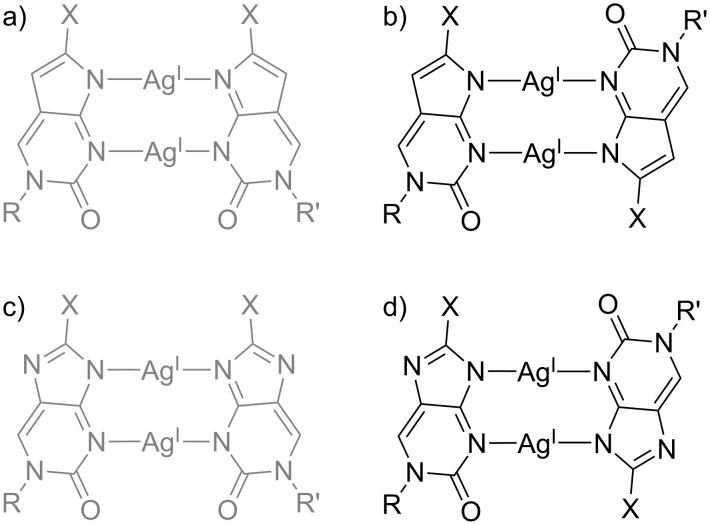
Possible base pairing patterns of dinuclear Ag(I)-mediated base pairs. Base pairs within an antiparallel-stranded context are displayed in grey, whereas base pairs in a parallel-stranded sequence alignment are shown in black. a, b) ^X^PC–Ag(I)_2_–^X^PC (X = phenyl, 2-pyridyl, 3-pyridyl) [62]; c, d) ^X^IC–Ag(I)_2_–^X^IC (X = H, phenyl, 2-furyl) [72]. R, R’ = DNA backbone.

In addition to engaging in base pairing with a complementary cytosine residue (vide supra), pyrrolocytosine derivatives were also investigated with respect to their propensity to form metal-mediated pyrrolocytosine:pyrrolocytosine base pairs [62]. These investigations not only included the ^2Pyr^PC and ^3Pyr^PC residues reported above, but also a ^Ph^PC nucleoside bearing a phenyl substituent. Accordingly, a possible additional stabilization due to the presence of the (potentially coordinating) endocyclic nitrogen atom of the pyridine substituent was investigated. A series of 12-mer duplexes and 25-mer duplexes were studied. In all cases, dinuclear Ag(I)-mediated base pairs formed (Scheme 7). Table 2 lists the thermal stabilization upon formation of the respective metal-mediated base pairs. As can be seen, base pairs including at least one ^2Pyr^PC moiety display the largest stabilization, which points towards an involvement of the endocyclic pyridyl nitrogen atom in metal coordination particularly for the parallel-stranded duplexes. For the shorter 12-mer duplexes, the stabilizing effect of metal-mediated base pair formation found in antiparallel-stranded DNA exceeds that observed in parallel-stranded DNA. For the longer 25-mer duplexes, the opposite is true, indicating the relevance of the sequence context on the observed stabilization. For all ^X^PC-derived base pairs, reversed Watson–Crick base pairing was enforced to ensure the formation of parallel-stranded duplexes.

**Table 2:** Increase in melting temperature Δ*T*_m_ of two sets of DNA duplexes bearing a ^X^PC–Ag(I)_2_–^Y^PC base pair (X, Y = phenyl, 2-pyridyl, 3-pyridyl) [62].

X	Y	Δ*T*_m_ [°C] for 12-mer duplex^a^			Δ*T*_m_ [°C] for 25-mer duplex^b^	
		
		aps^c^	ps^c^		aps^c^	ps^c^

2Pyr	2Pyr	+21.5	+21.0		+8.5	+13.5
3Pyr	3Pyr	+26.0	+10.0		+5.0	+7.0
Ph	2Pyr	+26.0	+14.5		+7.5	+12.0
Ph	3Pyr	+27.0	+5.5		n.d.	n.d.
3Pyr	2Pyr	+26.5	+19.0		+9.5	+13.0

^a^Parallel-stranded DNA obtained by using *^i^*G:C and G:*^i^*C base pairs; ^b^Parallel-stranded DNA with A:T base pairs, determined by the sequence only. ^c^aps: antiparallel-stranded (i.e., Watson–Crick base pairs, cisoid glycosidic bonds); ps: parallel-stranded (i.e., reversed Watson–Crick base pairs, transoid glycosidic bonds).

Formal replacement of one C–H group in pyrrolocytosine by a nitrogen atom leads to imidazolocytosine. A series of substituted imidazolocytosine (^X^IC) nucleobases were investigated with respect to their metal-binding properties, too [72]. In analogy to ^X^PC, ^X^IC forms dinuclear metal-mediated homo base pairs with Ag(I). Table 3 lists representative changes in the melting temperature upon formation of an ^X^IC–Ag(I)_2_–^X^IC base pair within a DNA duplex. As can be seen, these base pairs are extremely stabilizing both in antiparallel-stranded and in parallel-stranded duplexes. In fact, the ^2Fur^IC–Ag(I)_2_–^2Fur^IC pair represents the most stabilizing Ag(I)-mediated base pair reported to date. The trend in stabilization (^H^IC ≈ ^Ph^IC < ^2Fur^IC) allows two different explanations. As the furyl substituent is the only one with an potential donor atom [73], the extraordinary stability of the ^2Fur^IC–Ag(I)_2_–^2Fur^IC base pair may be the direct result of the formation of additional coordinate bonds. Alternatively (or in addition), the deprotonation of ^X^IC, which is a prerequisite for Ag(I) binding, is facilitated in the order ^H^IC < ^Ph^IC < ^2Fur^IC (p*K*_a_ = 8.8, 7.9, and 7.3 for the respective nucleosides), which is identical to the trend in stabilization [72]. For all ^X^IC-derived base pairs, parallel-stranded duplexes were obtained by enforcing reversed Watson–Crick base pairing.

**Table 3:** Increase in melting temperature Δ*T*_m_ of a representative DNA duplex bearing one ^X^IC–Ag(I)_2_–^X^IC base pair (X = H, phenyl, 2-furyl) [72].

Base pair	Δ*T*_m_ [°C]	

	aps^a^	ps^a^

^H^IC–Ag(I)_2_–^H^IC	+39.0	+27.0
^Ph^IC–Ag(I)_2_–^Ph^IC	+38.5	+27.0
^2Fur^IC–Ag(I)_2_–^2Fur^IC	+48.0	+38.0

^a^aps: antiparallel-stranded (i.e., Watson–Crick base pairs, cisoid glycosidic bonds); ps: parallel-stranded (i.e., reversed Watson–Crick base pairs, transoid glycosidic bonds).

**Scheme 8:**
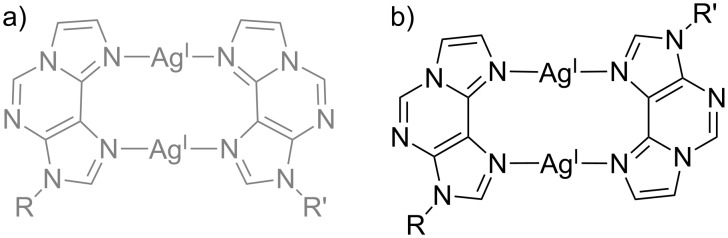
Base pairing patterns of the dinuclear Ag(I)-mediated homo base pair of 1,*N*^6^-ethenoadenine (εA) with a) cisoid arrangement of the glycosidic bonds (i.e., in an antiparallel-stranded duplex) [76] and b) transoid arrangement of the glycosidic bonds (i.e., in a parallel-stranded duplex) [75]. R, R’ = DNA backbone.

1,*N*^6^-Ethenoadenine (εA) is an exocyclic etheno adduct of adenine which was shown to bind transition metal ions better than its parent nucleobase [74]. Accordingly, its propensity to engage in metal-mediated base pairing was investigated in detail. As it turned out, εA is capable of simultaneously binding two metal ions with an almost parallel alignment of the N→M bonds [75]. In principle, both a cisoid and a transoid arrangement of the glycosidic bonds are feasible (Scheme 8). The former is adopted when the εA–Ag(I)_2_–εA base pair is incorporated in-between canonical Watson–Crick base pairs in a B-DNA duplex [76]. The stabilization observed upon formation of this cisoid εA–Ag(I)_2_–εA pair amounts to 12 °C. Interestingly, when the dinuclear [Ag_2_(εA)_2_]^2+^ complex is formed outside a DNA context, e.g., in a crystal structure using the model nucleobase 9-ethyl-1,*N*^6^-ethenoadenine or adsorbed onto HOPG applying 9-icosyl-1,*N*^6^-ethenoadenine (HOPG: highly-ordered pyrolytic graphite), the transoid conformation is preferred [75]. Accordingly, the transoid εA–Ag(I)_2_–εA base pair incorporated in-between reversed Hoogsteen base pairs in a parallel-stranded duplex brings about a stabilization of ≈16 °C, exceeding that of the cisoid pair. It should be noted that Hoogsteen (rather than reversed Hoogsteen) base pairing could not be ruled out completely for this parallel-stranded duplex, so that in principle a cisoid εA–Ag(I)_2_–εA base pair may also form when the complementary oligonucleotides are aligned in a parallel fashion. However, considering the intrinsic preference of εA–Ag(I)_2_–εA to adopt a transoid geometry and in line with the larger stabilization in a parallel-stranded duplex context, reversed Hoogsteen (Scheme 8b) represents the most likely base pairing pattern in this example [75].

**Scheme 9:**
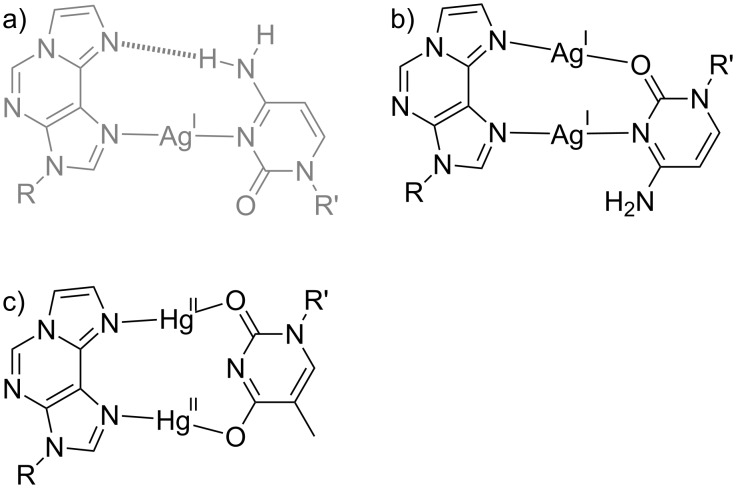
Additional metal-mediated base pairs involving 1,*N*^6^-ethenoadenine (εA). Base pairs within an antiparallel-stranded context are displayed in grey, whereas base pairs in a parallel-stranded sequence alignment are shown in black. a) εA–Ag(I)–C with a synergistic hydrogen bond [77]; b) εA–Ag(I)_2_–C [77]; c) εA–Hg(II)_2_–T [79–80]. R, R’ = DNA backbone.

Its property to align the N→M vectors in an almost parallel manner has led to the application of εA in a series of other metal-mediated base pairs. When locating a cytosine residue opposite εA, a fascinating influence of the relative strand orientation of the DNA duplex on the number of metal ions per base pair was observed [77]. When the εA:C pair is present inside an antiparallel-stranded duplex, it incorporates one Ag(I). The formation of the resulting εA–Ag(I)–C base pair (Scheme 9a) is accompanied by an increase in *T*_m_ of 15 °C. According to a geometry optimization of the proposed base pair structure, it also contains a synergistic hydrogen bond, as is evident from the non-linear N–Ag–N angle of 167°. Using the same sequence context, albeit with a parallel alignment of the strands with reversed Hoogsteen base pairing, a dinuclear εA–Ag(I)_2_–C pair is formed (Scheme 9b). The Ag···Ag distance within this base pair was calculated as 2.92 Å [77], suggesting the presence of a stabilizing argentophilic interaction [78]. The parallel-stranded duplex bearing an εA:C pair is stabilized by ≈20 °C upon incorporation of the two Ag(I) ions. This stabilization is not twice as large as that observed for the mononuclear base pair, indicating that the introduction of the second Ag(I) ion is only slightly more stabilizing than the synergistic hydrogen bond.

When a thymine residue is paired with εA in a parallel-stranded double helix with reversed Watson–Crick base pairs, then the resulting εA:T pair incorporates two Hg(II) ions, yielding εA–Hg(II)_2_–T as the first example of a dinuclear metal-mediated base pair bearing divalent metal ions [79]. In the proposed base pair structure, both Hg(II) ions are coordinated by an endocyclic nitrogen atom of εA and an exocyclic oxygen atom of the thymine residue [80]. This structure (Scheme 9c) differs slightly from the originally proposed one containing one additional bond from the endocyclic nitrogen atom of thymine to one of the Hg(II) ions, because a calculation of the Hg···N force constant [81] had resulted in an exceptionally low value of 0.7 N cm^−1^ [80]. A re-inspection of the originally proposed structure indicated that it represents a local energy minimum rather than the global one. In the structure shown in Scheme 9c, all Hg···N and Hg···O force constants amount to ≈2 N cm^−1^ and hence indicate strong bonds [80]. The fourfold positive charge of the two Hg(II) ions in the εA–Hg(II)_2_–T base pair is stabilized by three factors. First of all, the thymine residue is deprotonated upon coordination to Hg(II), as is also the case for the well-established T–Hg(II)–T base pair. Hence, the negative charge of the thyminate helps shielding the positive charge introduced by the metal ions. Second, the propensity of εA to bind two metal ions with the N→M bonds aligned in parallel brings together the Hg(II) ions at close distance. Third, the εA–Hg(II)_2_–T base pair appears to be formed in a parallel-stranded duplex only. Attempts to introduce it into an antiparallel sequence context were unsuccessful so far. The reason for this is unknown yet, but may be related to the transoid orientation of the glycosidic bonds in parallel-stranded DNA. The combination of these three effects thus allowed the formation of the first dinuclear metal-mediated base pair with two divalent metal ions.

## Conclusion

This review summarizes recent efforts to extend the principle of metal-mediated base pairing to parallel-stranded nucleic acid duplexes. It indicates the many experimental possibilities to enforce a parallel strand alignment. Depending on the requirements of the metal-mediated base pair to be formed, different strategies (e.g., using different pH values) can be followed. The exhaustive list of examples presented in this review allows drawing some general conclusions. The most important one probably is that in many cases it is not possible to predict a priori whether a metal-mediated base pair is more stabilizing in an antiparallel-stranded or a parallel-stranded duplex. The intrinsic stability of the metal-free duplex, its sequence and the method used to enforce a parallel orientation of the complementary strands play important roles, too. This becomes evident for example for the ^X^PC–Ag(I)_2_–^Y^PC base pairs, which were found to be more stabilizing in an antiparallel-stranded duplex for 12-mer oligonucleotides, whereas longer 25-mers showed a larger stabilization in a parallel-stranded orientation. Nonetheless, the use of parallel-stranded duplexes significantly extents the scope of metal-mediated base pairing, because it has been shown that artificial nucleobases can be designed in a way that they form metal-mediated base pairs that are more stabilizing in a parallel-stranded context than in an antiparallel-stranded one (e.g., FP–Ag(I)–FP, εA–Ag(I)_2_–C, ^2Fur^IC–Ag(I)_2_–^2Fur^IC). It is therefore beyond doubt that parallel-stranded DNA will find an important place in research on metal-mediated base pairs, in particular when the metal complex prefers a *C*_2_-symmetric geometry and hence a transoid orientation of the glycosidic bonds.
